# Proteasome Activation is Mediated via a Functional Switch of the Rpt6 C-terminal Tail Following Chaperone-dependent Assembly

**DOI:** 10.1038/srep14909

**Published:** 2015-10-09

**Authors:** Vladyslava Sokolova, Frances Li, George Polovin, Soyeon Park

**Affiliations:** 1 Department of Molecular, Cellular, and Developmental Biology University of Colorado, Boulder, CO 80309

## Abstract

In the proteasome, the proteolytic 20S core particle (CP) associates with the 19S regulatory particle (RP) to degrade polyubiquitinated proteins. Six ATPases (Rpt1-Rpt6) of the RP form a hexameric Rpt ring and interact with the heptameric α ring (α1–α7) of the CP via the Rpt C-terminal tails individually binding to the α subunits. Importantly, the Rpt6 tail has been suggested to be crucial for RP assembly. Here, we show that the interaction of the CP and Rpt6 tail promotes a CP-Rpt3 tail interaction, and that they jointly mediate proteasome activation via opening the CP gate for substrate entry. The Rpt6 tail forms a novel relationship with the Nas6 chaperone, which binds to Rpt3 and regulates the CP-Rpt3 tail interaction, critically influencing cell growth and turnover of polyubiquitinated proteins. CP-Rpt6 tail binding promotes the release of Nas6 from the proteasome. Based on disulfide crosslinking that detects cognate α3-Rpt6 tail and α2-Rpt3 tail interactions in the proteasome, decreased α3-Rpt6 tail interaction facilitates robust α2-Rpt3 tail interaction that is also strongly ATP-dependent. Together, our data support the reported role of Rpt6 during proteasome assembly, and suggest that its function switches from anchoring for RP assembly into promoting Rpt3-dependent activation of the mature proteasome.

The proteasome is an ATP-dependent protease responsible for regulated protein degradation in eukaryotes. The proteasome consists of a 28-subunit proteolytic core particle (CP) and 19-subunit regulatory particle (RP), which further divides into base and lid subassemblies^1^,^2^. The base contains six ATPases (Rpt1-Rpt6) that form a hetero-hexameric Rpt ring, and sits directly atop the CP. The lid laterally binds the base-CP complex to complete the proteasome holoenzyme. Upon the recognition of polyubiquitinated proteins, the lid cleaves polyubiquitin chains as the base unfolds and translocates the protein substrates into the CP, where peptide hydrolysis occurs^3^,^4^,^5^,^6^.

The CP consists of seven distinct α-type and β-type subunits that are arranged in a stack of four hetero-heptameric rings, α_1–7_-β_1–7_-β_1–7_-α_1–7_ 2,7. Three peptidase subunits (β1, β2 and β5) are concealed within the CP by the gate in the outer α ring to prevent unregulated degradation of cellular proteins^8^. In free CP, the gate is in a closed configuration via the N-termini of the seven α subunits that converge at the center of the α ring, plugging the substrate entry pore. The gate is in an open configuration in the proteasome holoenzyme, in which the outer α ring of the CP associates with the RP via seven inter-subunit ‘α pockets’ formed between neighboring α subunits^9^,^10^,^11^,^12^. These α pockets serve as docking sites for individual C-terminal tails of the six Rpt proteins. The occupation of α pockets by specific Rpt tails induces the opening of the gate^10^,^11^ and also mediates RP-CP assembly of the proteasome^13^,^14^,^15^,^16^,^17^. The Rpt tail-α interaction is stabilized via a salt bridge formed between the C-terminal carboxylate of the Rpt tail and the ε-amino group of the conserved lysine of the α subunit^9^.

The hetero-hexameric Rpt ring is arranged as Rpt3-Rpt6-Rpt2-Rpt1-Rpt5-Rpt4 in the proteasome^18^. Specifically, the C-terminal tails of Rpt3, Rpt2, and Rpt5 contain an evolutionarily conserved HbYX (hydrophobic amino acid-tyrosine-any amino acid) motif whose insertion into α pockets mediates CP gate opening^10^,^11^. Mutation of the HbYX motif, such as the deletion of the last amino acid or substitution of the tyrosine, decreases proteasome activities since incomplete opening of the gate suppresses substrate entry into the CP^11^, and sometimes compromises proteasome assembly^16^,^19^,^20^. Based on high-resolution structural studies of the proteasome holoenzyme, Rpt3, Rpt2, and Rpt5 tails are predominantly docked into their cognate α pockets^4^,^5^. These studies are consistent with the view that the proteasome exhibits optimal function and stability when a subset of Rpt tails dock into the CP^21^.

The hetero-hexameric Rpt ring assembles from three dimers, Rpt3-Rpt6, Rpt2-Rpt1, and Rpt5-Rpt4^18^,^22^,^23^. In each dimer, the C-domain proximal to the C-terminal tail of the Rpt proteins, binds to conserved chaperones, forming a pair-wise Rpt-chaperone interaction as follows: Rpt3-Nas6, Rpt6-Rpn14, Rpt1-Hsm3, and Rpt5-Nas2^23^,^24^,^25^,^26^. The binding of each chaperone to their cognate Rpt protein sterically clashes against the CP, blocking premature Rpt tail docking into the CP during assembly^17^,^26^,^27^. Recent studies provide further insights into this model, suggesting that chaperone-mediated regulation on its cognate Rpt tail may also involve a neighboring Rpt protein; Hsm3 scaffolds the Rpt1-Rpt2 dimer via binding to its cognate Rpt1 and the neighboring Rpt2^27^, and Nas2 binding to Rpt5 sterically clashes against Rpt1^18^,^28^. Whether such a trend is also observed in the Rpt3-Rpt6 dimer remains unknown.

At the hexameric Rpt ring-heptameric α ring interface of the proteasome, the Rpt3-Rpt6 dimer is positioned on the side of the Rpt ring where Rpt-α pocket interaction is specific as Rpt3-α2, Rpt6-α3, and Rpt2-α4, contrary to the other side of the ring where Rpt proteins bind multiple α pockets^29^. Importantly, the crucial role of the Rpt6 tail during Rpt ring assembly has been suggested to involve its exceptional specificity to its cognate α3 pocket^17^,^29^. When the tail peptides of the six Rpt proteins are mixed with purified CP, Rpt6 tail peptides uniquely insert into the cognate α3 pocket, whereas Rpt3 and Rpt2 tails show a marked preference for non-cognate pockets^17^. Thus, α3-Rpt6 tail interaction appears to act as an anchor that guides the rest of the α-Rpt tail interaction to establish the nascent CP-Rpt ring complex during assembly. However, the α3-Rpt6 tail interaction in the fully-formed proteasome is no longer predominant while its specificity remains intact^4^,^5^,^29^. This raises a question as to whether the Rpt6 tail has any functional role in the proteasome following assembly, and whether specific molecular events lead to an apparent change in CP-Rpt6 tail binding.

In the present study, we examined the role of Rpt6 tail binding to the CP in the proteasome. Our findings demonstrate that the CP-Rpt6 tail interaction is necessary for two functions that involve the CP-Rpt3 tail interaction in the proteasome holoenzyme: (1) CP gate opening for proteasome activation and (2) the release of the Nas6 chaperone to complete proteasome formation. The Rpt6 tail also exhibits a novel functional relationship with Nas6 *in vivo*. Importantly, our data demonstrate that the α3-Rpt6 tail interaction substantially decreases in the mature proteasome whereas α2-Rpt3 tail interaction increases. Our findings suggest that the Rpt6 tail undergoes a functional switch from mediating RP assembly^13^,^17^ into promoting Rpt3 tail-dependent opening of the gate to allow substrate entry into the mature proteasome.

## Results

### Rpt3 and Rpt6 jointly mediate proteasome activation via their C-terminal tail

**Figure 1:**
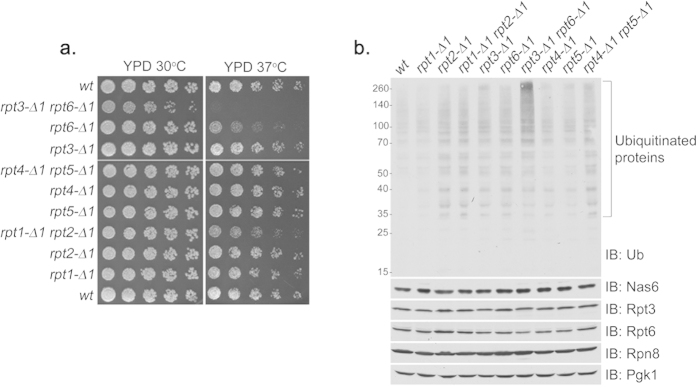
Together, the Rpt3 and Rpt6 tails play a central role in proteasome function. (**a**) Yeast growth assay showing severe heat sensitivity of *rpt3*-Δ*1rpt6*-Δ*1* double mutant cells. Four-fold serial dilutions of indicated yeast strains were spotted onto YPD plates and grown for 2–3 days at 30 °C and 37 °C. Table 1 lists the yeast strains used in each figure henceforth. (**b)** Anti-ubiquitin immunoblots showing an accumulation of polyubiquitinated proteins in *rpt3*-Δ*1rpt6*-Δ*1* cells. Levels of the Nas6 chaperone and proteasome subunits remained largely unchanged in all indicated strains. Whole cell lysates (20 μg) were subjected to 10% Bis-Tris SDS-PAGE for immunoblotting (IB) of polyubiquitinated proteins, and 12% Tris-Glycine SDS-PAGE (SDS-PAGE henceforth) for immunoblotting of Nas6 and proteasome subunits. Nas6 is a cognate chaperone of Rpt3. Rpt3 and Rpt6 are base subunits. Rpn8 is a lid subunit. Pgk1 serves as a loading control. Ub is ubiquitin. Molecular weight markers are at left in kDa.

To examine whether the interaction in each Rpt dimer, Rpt3-Rpt6, Rpt4-Rpt5, and Rpt1-Rpt2, is relevant to the function of each Rpt tail binding to the CP, we constructed pair-wise Rpt mutant strains in *S. cerevisiae*, *rpt3*-Δ*1rpt6*-Δ*1*, *rpt4*-Δ*1rpt5*-Δ*1*, and *rpt1*-Δ*1rpt2*-Δ*1* from individual *rpt*-Δ*1* strains, in which each Rpt subunit lacks a single C-terminal amino acid for CP binding (Fig. 1a). To distinguish which specific Rpt pair is particularly important for proteasome function, we examined the growth phenotypes of these mutants. The *rpt3*-Δ*1rpt6*-Δ*1* double mutants already displayed a mild growth defect under normal conditions at 30 °C on YPD medium compared to all other mutants. The growth impairment of the *rpt3*-Δ*1rpt6*-Δ*1* double mutants was exacerbated at the elevated temperature of 37 °C (Fig. 1a). Consistent with the crucial role of the Rpt6 tail in RP assembly, *rpt6*-Δ*1* cells have been shown to be hyper-sensitive to heat stress^13^,^14^,^17^. However, it was unexpected that the *rpt3*-Δ*1rpt6*-Δ*1* double mutants exhibited further growth impairment at both 30 °C and 37 °C, since *rpt3*-Δ*1* cells alone appeared nearly indistinguishable from wild-type (Fig. 1a). The other two pairs of Rpt mutants, *rpt4*-Δ*1rpt5*-Δ*1* and *rpt1*-Δ*1rpt2*-Δ*1* displayed no apparent defect at 30 °C and only mild growth impairment at 37 °C.

To corroborate whether impaired growth in *rpt3*-Δ*1rpt6*-Δ*1* double mutant cells results from deficient proteasome function *in vivo*, we examined the level of polyubiquitinated proteins. Indeed, the *rpt3*-Δ*1rpt6*-Δ*1* cells severely accumulated polyubiquitinated proteins compared to all other cells (Fig. 1b), suggesting that their proteasomes are functionally defective. The levels of proteasome subunits in these cells remained largely comparable to wild-type (Fig. 1b), confirming that the single amino acid deletion from each Rpt tail does not change the levels of individual subunits. The *rpt1*-Δ*1rpt2*-Δ*1* and *rpt4*-Δ*1rpt5*-Δ*1* cells did not noticeably accumulate polyubiquitinated proteins (Fig. 1b). Together, these data suggest that Rpt3 and Rpt6 jointly play a central role in proteasome function via their C-terminal tail binding to the CP.

**Figure 2:**
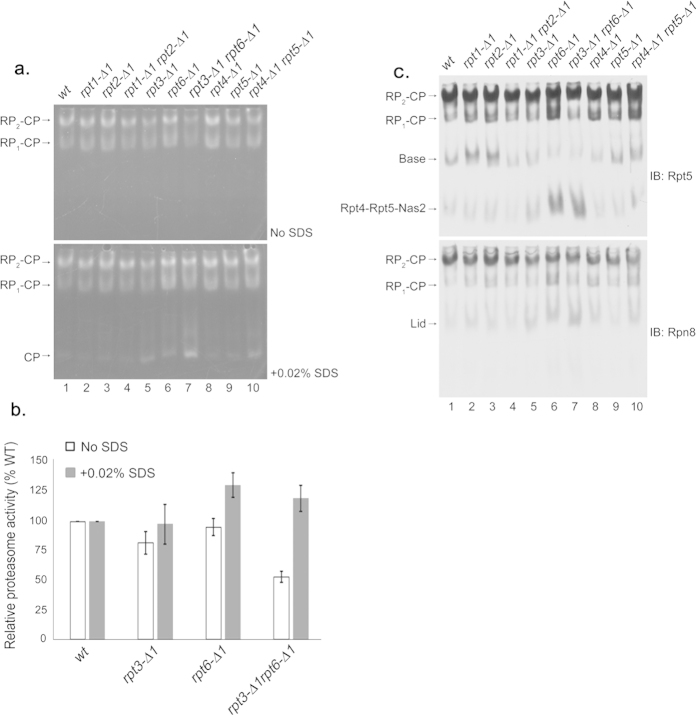
Rpt6 tail binding to the CP is crucial for Rpt3 tail-mediated gate opening in the proteasome holoenzyme. (**a**) Decreased proteasome activity in *rpt3*-Δ*1rpt6*-Δ*1* cells (top panel, RP_2_-CP and RP_1_-CP) is restored upon artificial opening of the CP gate (bottom panel, RP_2_-CP and RP_1_-CP). Whole cell lysates (80 μg) from indicated strains were resolved by 3.5% native PAGE. The native gel was incubated with fluorogenic peptide substrate LLVY-AMC to visualize the proteasomes (top panel). Following the imaging of the native gel, the same gel was further incubated with LLVY-AMC in the presence of 0.02% SDS (bottom panel). The addition of SDS is known to open the CP gate^8^. RP_2_-CP and RP_1_-CP are doubly-capped and singly-capped proteasome holoenzymes, respectively. The CP gate within wild-type proteasome holoenzymes is in an open configuration and is not further enhanced by 0.02% SDS^8^. (**b**) Quantification of relative proteasome activities (RP_2_-CP and RP_1_-CP) from seven independent experiments as in (**a**) plotted as mean ± standard error of the mean (SEM). Values of proteasome activities of the indicated strains were quantified using ImageJ software, and normalized to wild-type to obtain the relative proteasome activities. Calculations were performed individually for samples in the absence of SDS as in top panel from (**a**), and in the presence of 0.02% SDS as in bottom panel from (**a**) for each experiment. (**c**) Impaired RP assembly and defective proteasome holoenzymes in *rpt3*-Δ*1rpt6*-Δ*1* cells. Following proteasome activity assays with the LLVY-AMC as in (**a**), the native gels were subjected to immunoblotting (IB) to detect the proteasome holoenzymes and their subassemblies using antibodies to a base subunit Rpt5 (top panel), and a lid subunit Rpn8 (bottom panel).

To understand the basis of impaired proteasome function in the *rpt3*-Δ*1rpt6*-Δ*1* double mutant cells, we used nondenaturing gel electrophoresis (native PAGE henceforth) and in-gel peptidase assays using the fluorogenic peptide substrate LLVY-AMC^30^ to assess the overall activity and assembly state of the proteasome from whole cell lysates (Fig. 2a). The Rpt3 tail contains the HbYX motif that is essential for gate opening in the proteasome^11^. In *rpt3*-Δ*1* cells, activities of the proteasome holoenzymes appeared slightly decreased as compared to wild-type (Fig. 2a, top, RP_2_-CP and RP_1_-CP), indicating the blockage of substrate entry due to defects in gate opening^11^. Consistent with the lack of HbYX motif in the Rpt6 tail, proteasome activities of the *rpt6*-Δ*1* cells were comparable to wild-type. However, the *rpt3*-Δ*1rpt6*-Δ*1* double mutant cells exhibited a noticeable decrease in their proteasome activities (Fig. 2a, top, RP_2_-CP and RP_1_-CP), which are estimated to be only 50% of wild-type based on our quantification (Fig. 2b). To examine whether reduced activity of the proteasomes in the *rpt3*-Δ*1rpt6*-Δ*1* cells results from defects in gate opening, we added 0.02% SDS that is known to artificially open the gate^8^,^11^. Importantly, proteasome activities in *rpt3*-Δ*1rpt6*-Δ*1* cells returned to normal upon the addition of the SDS (Fig. 2a, bottom, RP_2_-CP and RP_1_-CP, and Fig. 2b), suggesting that these proteasomes are severely compromised in gate opening. Also, proteasome activities in *rpt6*-Δ*1* cells further increased as compared to wild-type upon the addition of SDS, indicating that CP-Rpt6 tail interaction might serve a regulatory role to prevent aberrant enhancement of gate opening in the proteasome. Proteasome activities in *rpt1*-Δ*1rpt2*-Δ*1* and *rpt4*-Δ*1rpt5*-Δ*1* cells remained largely comparable to wild-type despite the presence of HbYX motif in Rpt2 and Rpt5 (Fig. 2a and Supplementary Fig. S1 online). Therefore, Rpt3 and Rpt6 tails jointly regulate gate opening for proper activation of the proteasome.

Given the central role of Rpt6 tail binding to the CP during RP assembly^13^,^17^, we examined the assembly states of the proteasome holoenzyme and its RP assembly intermediates via immunoblotting of native gels. The level of the proteasomes in *rpt3*-Δ*1rpt6*-Δ*1* cells was comparable to wild-type (Fig. 2c), confirming that reduced proteasome activity in these cells results from defective gate opening (Fig. 2a,b) and not from decreased proteasome levels. Importantly, the *rpt3*-Δ*1rpt6*-Δ*1* cells displayed indications of deregulated RP assembly as evident from increased level of Rpt4-Rpt5-Nas2, a representative base assembly intermediate, and concurrently decreased level of free base (Fig. 2c, top, lane 7). Free lid also increased in *rpt3*-Δ*1rpt6*-Δ*1* cells (Fig. 2c, bottom, lane 7). These defects in assembly appeared largely comparable between *rpt6*-Δ*1* and *rpt3*-Δ*1rpt6*-Δ*1* cells (Fig. 2c, lanes 6, 7). The *rpt3*-Δ*1* cells alone did not show these defects. Therefore, impaired Rpt6 tail interaction with the CP mainly accounts for the perturbation in RP assembly. RP assembly intermediates in *rpt1*-Δ*1rpt2*-Δ*1* and *rpt4*-Δ*1rpt5*-Δ*1* cells did not change as substantially as in *rpt3*-Δ*1rpt6*-Δ*1* cells. Together, our findings indicate that successful interaction of the Rpt6 tail with the CP may serve as a regulatory mechanism to promote proper Rpt3 tail binding to the CP to open the gate in the proteasome holoenzyme.

### Both Rpt6 and Rpt3 tails are required for the eviction of Nas6 upon proteasome assembly

**Figure 3:**
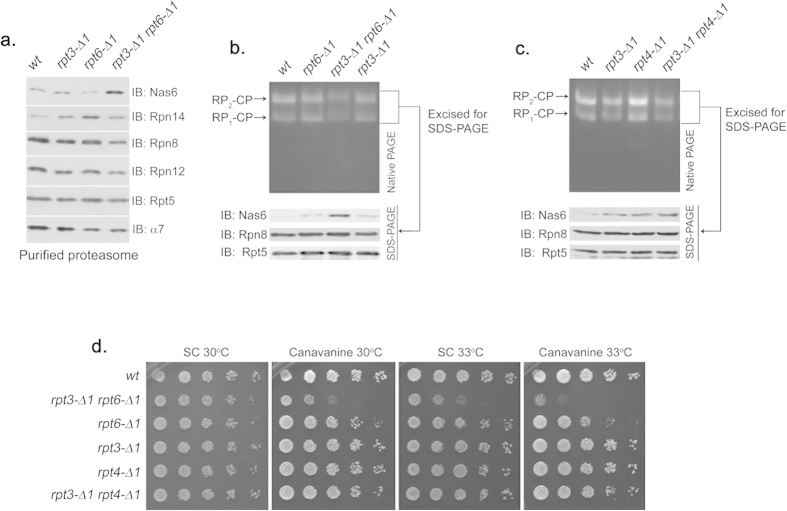
Rpt6 tail binding to the CP is required for Rpt3 tail-mediated release of Nas6 from the proteasome holoenzyme. (**a**) Increased retention of Nas6 in the proteasomes purified from *rpt3*-Δ*1rpt6*-Δ*1* cells. Proteasomes were isolated from indicated strains via a Protein A tag that is appended to CP subunit Pre1^44^. Purified proteasomes (1 μg) were subjected to 12% SDS-PAGE and immunoblotting (IB) for the indicated proteins: Nas6, Rpn14, chaperones; Rpn8, Rpn12, lid subunits; Rpt5, base subunit; α7, CP subunit. Pre1-TEV-ProA tag was chosen to exclude the purification of free RP. Note that Nas6 abundance remains unchanged in these cells (Fig. 1b). (**b**) Increased retention of Nas6 in the proteasomes in whole cell extracts from *rpt3*-Δ*1rpt6*-Δ*1* cells. Whole cell lysates (40 μg) from indicated strains were resolved by 3.5% native gel (top panel). Following proteasome activity assay with LLVY-AMC, the native gel region containing the proteasome holoenzymes (RP_2_-CP and RP_1_-CP) was excised into a horizontal strip and directly subjected to 12% SDS-PAGE and immunoblotting for indicated proteins (bottom panel). Rpn8 (a lid subunit) and Rpt5 (a base subunit) serve as loading controls for proteasome levels in the native gel strip. Note that decreased proteasome activities in *rpt3*-Δ*1rpt6*-Δ*1* cells are due to gate opening defects (Fig. 2a,b). (**c**) Nas6 level in the proteasomes from *rpt3*-Δ*1rpt4*-Δ*1* cells is comparable to that in the proteasomes from *rpt3*-Δ*1* cells. Experiments were carried out as in (**b**) to assess the level of Nas6 in the proteasome holoenzymes from indicated cells. Rpn8 (a lid subunit) and Rpt5 (a base subunit) serve as loading controls for proteasome levels in the native gel strip. (**d**) Phenotypic analysis of indicated yeast strains showing that the Rpt3 tail exhibits stronger genetic interaction with the Rpt6 tail than the Rpt4 tail. Four-fold serial dilutions of the indicated cells were spotted onto synthetic complete medium (SC) and grown for 2–3 days at 30 °C and 33 °C. For testing sensitivity to canavanine (an arginine analog), arginine was omitted from the SC medium. Canavanine was included at 1 μg/ml final concentration.

During RP assembly, Rpt3 and Rpt6 bind to their cognate chaperone, Nas6 and Rpn14, respectively^22^,^23^,^26^,^31^. Docking of the Rpt tail into the CP appears to displace their cognate chaperone^13^,^17^,^26^, providing an explanation for their absence on the fully-formed proteasome. When the Rpt3 tail is specifically perturbed in interacting with the CP as in *rpt3*-Δ*1* cells, it fails to displace Nas6, resulting in Nas6-containing proteasomes^13^. Likewise, proteasomes from *rpt6*-Δ*1* cells aberrantly retain Rpn14^13^. To further understand the influence of the Rpt6 tail on the CP-Rpt3 tail interaction, we examined the presence of their cognate chaperones on the proteasomes from *rpt3*-Δ*1rpt6*-Δ*1* double mutant cells. We isolated the proteasome holoenzymes using a Protein A tag appended to CP subunit Pre1 (β4) to exclude purification of free RP that is in complex with the chaperones^13^,^23^,^26^,^31^. Rpn14 level was elevated on the *rpt6*-Δ*1* proteasome, and Nas6 on the *rpt3*-Δ*1* proteasome (Fig. 3a), consistent with previous findings^13^. Importantly, the proteasomes from *rpt3*-Δ*1rpt6*-Δ*1* double mutant cells retained an even greater amount of Nas6 while Rpn14 apparently released (Fig. 3a). The level of Nas6 remained constant in whole cell lysates regardless of Rpt tail mutation (Fig. 1b), verifying that Nas6 enrichment on the *rpt3*-Δ*1rpt6*-Δ*1* proteasome results from failed release of Nas6. Therefore, our findings suggest that the complete release of Nas6 from the proteasome requires its cognate Rpt3 tail as well as the Rpt6 tail to successfully interact with the CP although the Rpt6 tail does not directly interact with Nas6^23^,^26^.

To rule out the possibility that the purification procedure might lead to an artificial enrichment of Nas6 in the *rpt3*-Δ*1rpt6*-Δ*1* proteasome, we resolved the proteasomes directly from whole cell lysates by native PAGE without performing affinity-purification. First, we visualized proteasome holoenzymes (RP_2_-CP and RP_1_-CP) on the native gel by in-gel peptidase assay (Fig. 3b, top). Then, we excised the native gel region that contains both species of the proteasome (RP_2_-CP and RP_1_-CP), and resolved the native gel strip directly by SDS-PAGE to detect Nas6 on the proteasome (Fig. 3b, bottom, See Methods). Consistent with our data based on affinity-purified proteasomes (Fig. 3a), the proteasome holoenzymes from the *rpt3*-Δ*1rpt6*-Δ*1* cells contained an increased level of Nas6, as compared to the proteasomes from *rpt3*-Δ*1* or *rpt6*-Δ*1* cells (Fig. 3b).

To confirm that the Rpt6 tail specifically influences Rpt3 tail-mediated eviction of Nas6 from the proteasome, we constructed an additional double mutant strain, *rpt3*-Δ*1rpt4*-Δ*1* since Rpt4 is the other direct neighbor of Rpt3, but does not form a dimer with Rpt3 on the hetero-hexameric Rpt ring in the proteasome^18^,^22^,^23^. If the CP-Rpt4 tail interaction is important for CP-Rpt3 tail interaction, Nas6 is expected to aberrantly remain on the *rpt3*-Δ*1rpt4*-Δ*1* proteasomes at a greater level than on the *rpt3*-Δ*1* proteasomes. To examine the level of Nas6 on the proteasome holoenzymes from the *rpt3*-Δ*1rpt4*-Δ*1* cells, we directly resolved whole cell lysates from these cells using native PAGE and the second dimension SDS-PAGE as above (Fig. 3c). Nas6 was detectable on the *rpt4*-Δ*1* proteasomes, likely due to the role of Rpt4 tail in proteasome assembly^13^. However, the level of Nas6 on the *rpt3*-Δ*1rpt4*-Δ*1* proteasomes remained largely comparable to the *rpt3*-Δ*1* proteasomes, indicating that Rpt4 tail docking onto the CP does not substantially influence Rpt3 tail docking to evict Nas6 from the proteasome. Also, the growth of *rpt3*-Δ*1rpt4*-Δ*1* cells was comparable to wild-type, contrary to the severe growth impairment of *rpt3*-Δ*1rpt6*-Δ*1* cells (Fig. 3d). Our data suggest that Rpt6 tail binding to the CP is specifically required for the interaction of the Rpt3 tail with the CP to fully evict Nas6 from the proteasome upon completion of assembly.

### A novel functional relationship between the Rpt6 tail and the Nas6 chaperone

**Figure 4:**
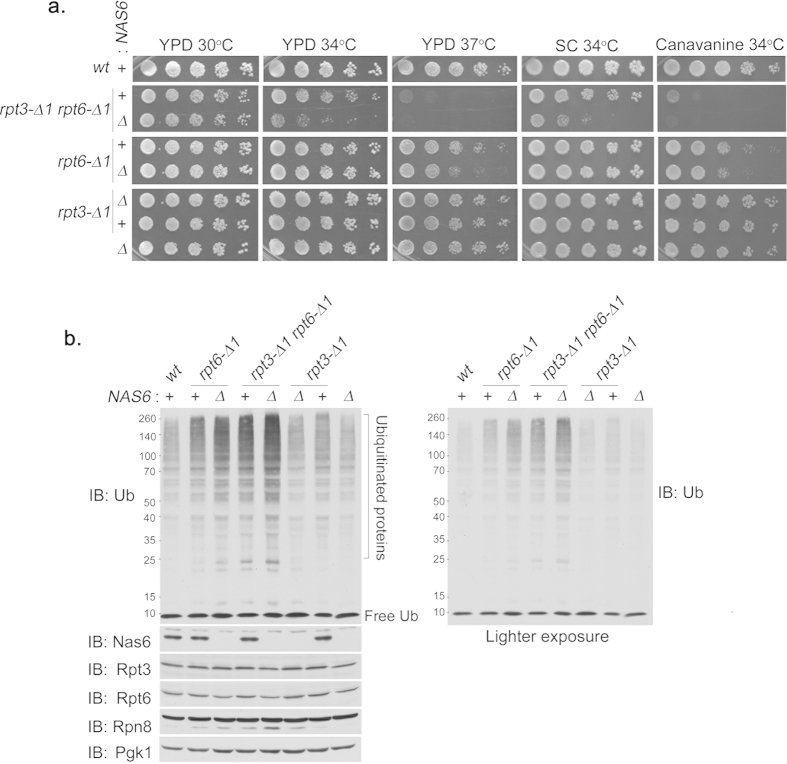
The Rpt6 tail exhibits a distinct functional relationship with Nas6 *in vivo*. (**a**) Phenotypic analysis showing effect of *nas6*Δ on the growth of *rpt6*-Δ*1* or *rpt3*-Δ*1* single, or double mutants. Four-fold serial dilutions of indicated cells were spotted onto YPD plates, synthetic complete medium (SC), or SC medium containing canavanine (1 μg/ml), and incubated for 2–3 days at the indicated temperature. For testing sensitivity to canavanine (an arginine analog), arginine was omitted from the SC medium. (**b**) Effect of *nas6*Δ on the degradation of polyubiquitinated proteins in *rpt6*-Δ*1* or *rpt3*-Δ*1* single, or double mutant cells. The cells were cultured for 6 hours at 37 °C. Whole cell lysates (20 μg) were subjected to 10% Bis-Tris SDS-PAGE for immunoblotting (IB) of polyubiquitinated proteins, and 12% SDS-PAGE for immunoblotting of Nas6 and proteasome subunits. Rpt3 and Rpt6 are base subunits. Rpn8 is a lid subunit. Pgk1 serves as a loading control. Lighter exposure of anti-ubiquitin (Ub) immunoblot is shown at right to further illustrate the difference in polyubiquitinated protein levels. Molecular weight markers are at left in kDa.

Based on the striking enrichment of Nas6 on the *rpt3*-Δ*1rpt6*-Δ*1* proteasomes (Fig. 3a,b), one can make two alternative predictions. First, Nas6 reflects the docking status of the Rpt3 tail into the CP without directly contributing to proteasome assembly. Second, Nas6 plays a distinct role in proteasome assembly together with the Rpt3 and Rpt6 tails. To examine these possibilities, we assessed the phenotypes of Rpt3 and Rpt6 tail mutants with or without Nas6. Even under mild heat stress at 34 °C, the *rpt3*-Δ*1rpt6*-Δ*1nas6*Δ triple mutants exhibited severe growth defects as compared to *rpt3*-Δ*1rpt6*-Δ*1* cells (Fig. 4a). At 37 °C, the *rpt3*-Δ*1rpt6*-Δ*1nas6*Δ cells were nearly lethal. Similarly, in the presence of canavanine, an arginine analog that increases the flux of misfolded proteins to the proteasome, the growth defect of the *rpt3*-Δ*1rpt6*-Δ*1nas6*Δ cells was more severe than the *rpt3*-Δ*1rpt6*-Δ*1* cells (Fig. 4a). Nas6-dependent effects were also evident in *rpt6*-Δ*1* single mutants since *rpt6*-Δ*1nas6*Δ double mutants showed increased sensitivity to heat stress at 37 °C and to canavanine at 34 °C (Fig. 4a). These data suggest a novel functional relationship between Rpt6 and Nas6. When the Rpt6 tail is intact, *nas6*Δ, *rpt3*-Δ*1*, and *rpt3*-Δ*1nas6*Δ cells grow like wild-type under our conditions, indicating that Rpt6 tail binding to the CP could apparently compensate for the loss of Nas6 or the Rpt3 tail, singly and in combination. In line with these results, *NAS6* overexpression did not appreciably affect the growth of *rpt3*-Δ*1, rpt6*-Δ*1* or *rpt3*-Δ*1rpt6*-Δ*1* cells (Supplementary Fig. S2 online), indicating that increased dosage of *NAS6* cannot substitute the role of Rpt tail binding to the CP during proteasome assembly.

To ascertain whether Nas6-dependent effects on cell growth result from deficient proteasome function, we examined turnover of cellular polyubiquitinated proteins. The levels of polyubiquitinated proteins in *nas6*Δ and *rpt3*-Δ*1nas6*Δ cells were similar to wild-type (Fig. 4b), consistent with their largely normal growth phenotypes (Fig. 4a). Contrarily, polyubiquitinated proteins noticeably accumulated in *rpt6*-Δ*1*, and further increased in *rpt6*-Δ*1nas6*Δ cells (Fig. 4b), confirming the functional relationship between Nas6 and the Rpt6 tail (Fig. 4a). Similarly, the level of polyubiquitinated proteins in *rpt3*-Δ*1rpt6*-Δ*1* double mutants increased in the *rpt3*-Δ*1rpt6*-Δ*1nas6*Δ triple mutant cells (Fig. 4b). Together, these results suggest a coordinated role of the Rpt6 tail, Rpt3 tail, and Nas6 in the regulation of proteasome *in vivo*.

**Figure 5:**
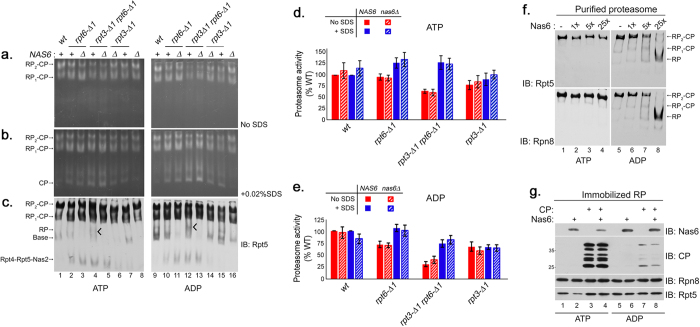
Nas6 maintains the abundance of RP species during Rpt tail-CP interaction. (**a**,**b**) Nas6 is not directly responsible for gate opening defects in the proteasome from *rpt3*-Δ*1rpt6*-Δ*1* cells. Whole cell lysates (80 μg) were prepared in the presence of ATP (left) or ADP (right) at 2 mM each, and subjected to 3.5% native PAGE. In-gel peptidase assays with LLVY-AMC were performed to assess the gate opening in the proteasome holoenzymes in the absence or presence of 0.02% SDS. (**c**) Nas6 maintains intracellular levels of RP and base species. The native gels as in (**a**,**b**) were subjected to immunoblotting (IB) to detect proteasome holoenzymes and their subassemblies using an antibody to a base subunit Rpt5. RP species become prominent in *rpt3*-Δ*1rpt6*-Δ*1* cells under ADP condition (tick mark, lanes 4, 12). (**d,e**) Proteasome activities (RP_2_-CP and RP_1_-CP) of the indicated strains from (**a**,**b**) were quantified using ImageJ and normalized to wild-type (% WT) as mean ± SEM (n = 6, ATP; n = 4, ADP). Calculations were performed individually for samples in the absence of SDS, and in the presence of 0.02% SDS. (**f**) Nas6 promotes RP dissociation from the proteasome in the presence of ADP. Proteasomes were purified with a Protein A tag appended to a lid subunit Rpn11^44^. Affinity-purified proteasomes (0.6 pmol) were incubated with 1, 5, and 25 fold molar excess of recombinant Nas6 for 30 min at 30 °C in the presence of ATP (2 mM) or ADP (2 mM). The samples were subjected to 3.5% native PAGE and immunoblotting for a base subunit Rpt5 and a lid subunit Rpn8 to detect proteasome holoenzymes and RP species. (**g**) Nas6 remains bound to the RP when RP-CP interaction is destabilized by ADP. RP was affinity-purified using Rpn11-TEV-ProA from the *nas6*Δ strain and was left on IgG resin. Immobilized *nas6*Δ RP (5 μl) was incubated with purified CP (3 μg) or recombinant Nas6 (0.5 μg), singly and in combination for 30 min at 30 °C, with either ATP or ADP (2 mM). RP-bound material was washed, eluted with TEV protease, and analyzed via 12% SDS-PAGE and immunoblotting.

To investigate whether Nas6 influences the joint function of the Rpt6 and Rpt3 tails on proteasome activation or assembly (Figs 2 and 3), we first analyzed potential effects of Nas6 on gate opening via the Rpt6 and Rpt3 tails, using the proteasome activity assay with the fluorogenic substrate LLVY-AMC (Fig. 5a). The entry of LLVY-AMC into the CP via gate opening is rate limiting for LLVY-AMC hydrolysis by the proteasome^8^. Also, it is well established that the RP stimulates CP peptidase activity via gate opening as the HbYX-type Rpt tails dock into their cognate α pockets and induce an open configuration of the substrate entry pore in the α ring^9^,^10^,^11^, rather than allosterically activating the peptidase subunits (β1, β2, β5). Therefore, the proteasome activity assay can be used to assess the status of gate opening^8^,^9^,^11^,^14^. We visualized the activities of proteasome holoenzymes from the mutants from Fig. 4 via native PAGE and in-gel peptidase assay (Fig. 5a). Whether or not Nas6 was present, the activities of the proteasome holoenzymes appeared largely comparable between *rpt6*-Δ*1* and *rpt6*-Δ*1nas6*Δ cells, and also between *rpt3*-Δ*1rpt6*-Δ*1* and *rpt3*-Δ*1rpt6*-Δ*1nas6*Δ cells (Fig. 5a, left, RP_2_-CP and RP_1_-CP; see Fig. 5d for quantification). Consistent with our results in Fig. 2, gate opening defects in *rpt3*-Δ*1rpt6*-Δ*1* proteasomes were rescued upon the addition of 0.02% SDS, and was not affected by Nas6 (Fig. 5b, left; see Fig. 5d for quantification). These data indicate that gate opening in the proteasome holoenzyme is largely dependent on the Rpt3 and Rpt6 tails, and is not directly mediated by Nas6.

To further probe Nas6-mediated effects on the proteasome from *rpt3*-Δ*1rpt6*-Δ*1* cells, we prepared whole cell lysates in the presence of ADP, which generally destabilizes the Rpt tail interaction with the CP of the proteasome^32^ (Fig. 5a,b, right). Proteasome activities diminished in both *rpt3*-Δ*1rpt6*-Δ*1* and *rpt3*-Δ*1rpt6*-Δ*1nas6*Δ cells relative to wild-type, indicating the importance of Rpt3 and Rpt6 tails in their nucleotide-dependent interaction with the CP (Fig. 5a,b, lanes 12, 13; see Fig. 5e for quantification). However, proteasome activities appeared mostly similar between *rpt3*-Δ*1rpt6*-Δ*1* and *rpt3*-Δ*1rpt6*-Δ*1nas6*Δ cells (Fig. 5a,b, lanes 12, 13; also see Fig. 5e for quantification), supporting our conclusion above that Nas6 does not directly control gate opening in the proteasome.

Next, we tested whether the relationship between Nas6 and the Rpt6 tail is important for proteasome assembly. We examined the abundance of RP assembly intermediates, which sensitively report the overall status of proteasome assembly in cells even when the changes in the mature proteasome levels are subtle (Fig. 5c). Immunoblotting of the native gels revealed that RP and base species significantly decreased whenever Nas6 was absent, and this trend became more prominent in the presence of ADP (Fig. 5c). In particular, *rpt3*-Δ*1rpt6*-Δ*1* cells exhibited an additional RP-like species that disappeared in the absence of Nas6 (Fig. 5c, tick mark, lanes 4, 12). Given that Nas6 is in complex with RP and base^13^,^23^,^31^ and that proteasome assembly involves nucleotide-dependent Rpt tail-CP interaction^17^,^19^,^20^, our data indicate that the roles of Nas6 include maintaining the abundance of RP assembly intermediates during proteasome assembly.

To corroborate whether Nas6-RP complex could reflect the changes in the CP-Rpt tail interaction of the proteasome, we isolated the proteasomes from wild-type cells and added Nas6 in an increasing amount under ATP and ADP conditions (Fig. 5f). In the presence of ATP, the proteasomes remained stable even at 25-fold molar excess of Nas6. In the presence of ADP, the proteasomes decreased as the free RP concurrently increased when Nas6 was added at 5-fold and 25-fold molar excess (Fig. 5f, lanes 7, 8). Rpn14, the cognate chaperone for Rpt6, did not promote RP dissociation from the proteasome even at 25-fold molar excess in the presence of ADP (Supplementary Fig. S3 online). Thus, ADP-dependent increase in Nas6-RP complex may reflect nucleotide-dependent interaction of its cognate Rpt3 tail with the CP.

Next, we examined how RP-CP interaction affects RP-Nas6 interaction in a nucleotide-dependent manner. We immobilized RP, and added purified CP or Nas6 singly and in combination (Fig. 5g). Under ATP, the RP-CP complex readily formed (Fig. 5g, lanes 3, 4). When Nas6 was added to the RP, the RP-CP complex formed upon displacing Nas6 as indicated by decreased Nas6 on the RP (Fig. 5g, lane 4). Under ADP, RP-CP complex formation dramatically diminished as compared to ATP condition, and failed to evict Nas6 from the RP (Fig. 5g, lane 8). These results support that nucleotide-dependent RP-CP interaction influences the level of Nas6-RP complexes.

### Rpt6 and Rpt3 jointly regulate the proteasome holoenzyme via decreased α3-Rpt6 tail interaction and increased α2-Rpt3 tail interaction

**Figure 6:**
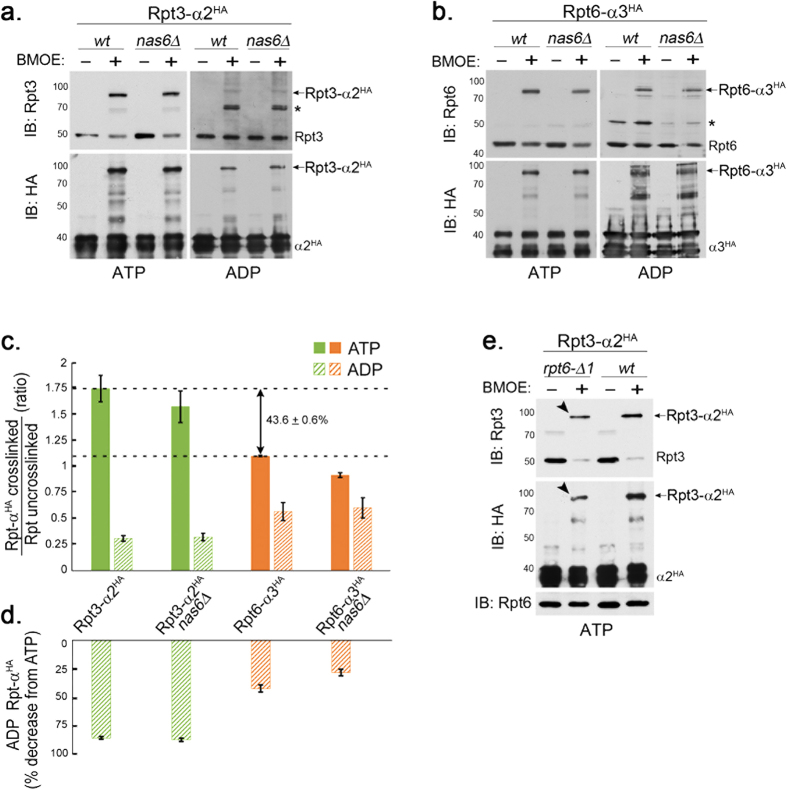
The Rpt6 tail-α3 interaction decreases in the proteasome holoenzyme and is less nucleotide-dependent than the Rpt3 tail-α2 interaction. (**a**) Rpt3 tail-α2 binding is strongly ATP-dependent in the proteasome holoenzyme, but is Nas6-independent. Wild-type and *nas6*Δ cells each harbor *rpt3-K428C* and *α2-A79C-HA*_*6*_ alleles; K428 is the last residue of Rpt3. Proteasomes were immunoprecipitated via α2-HA_6_ in the presence of ATP (1 mM) or ADP (2 mM), and then incubated with a chemical crosslinker BMOE (0.1 mM), or its solvent, DMF for 1 hour at 4 °C and subjected to SDS-PAGE. Rpt3-α2^HA^ crosslinks were detected by immunoblotting (IB) for Rpt3 and α2^HA^. Molecular weight markers are at left in kDa. Asterisk (*) indicates a non-specific signal. Crosslinked products remain stable during our analysis since BMOE is an irreversible crosslinker. (**b**) Rpt6 tail-α3 binding occurs in both ATP and ADP in the proteasome holoenzyme. Experiments were conducted as in (**a**) in wild-type and *nas6*Δ cells, each harboring *rpt6-K405C* and *α3-T81C-HA*_*6*_ alleles; K405 is the last residue of Rpt6. Rpt6-α3^HA^ crosslinks were detected by immunoblotting for Rpt6 and α3^HA^. Asterisk (*) indicates a non-specific band. (**c**) Rpt6-α3 interaction decreases in the proteasome holoenzyme whereas Rpt3-α2 interaction increases. The Rpt3-α2 crosslinks and Rpt6-α3 crosslinks as in (**a**,**b**) were quantified using ImageJ software and shown as mean + SEM (n = 6, Rpt3-α2, ATP; n = 3, Rpt3-α2, ADP and Rpt6-α3, ATP; n = 4, Rpt6-α3, ADP). The ratio on the Y axis was obtained by normalizing the intensities of Rpt-α^HA^ crosslinked bands to corresponding uncrosslinked Rpt bands on the same immunoblot, for example, [Rpt6-α3^HA^ band (80 kDa)]/[Rpt6 band (45 kDa)] (Fig. 6b, lane 2, top). Note that 43.6 + 0.6% decrease in Rpt6-α3^HA^ ratio (ATP) is relative to Rpt3-α2^HA^ ratio (ATP). (**d**) Rpt6-α3 interaction occurs in ADP whereas Rpt3-α2 interaction severely decreases. The Y axis indicates percent decrease of Rpt-α^HA^ ADP samples relative to their corresponding ATP samples from (**c**). (**e**) The Rpt6 tail is crucial for Rpt3-α2 binding (arrow heads) in the proteasome holoenzyme. Experiments were conducted as in (**a**) in *rpt6*-Δ*1* and wild-type cells, each carrying *rpt3-K428C* and *α2-A79C-HA*_*6*_ alleles. Rpt6 levels remain unchanged.

Our results above suggest that nucleotide-dependent CP-Rpt tail interaction is an important feature of the Rpt proteins, and does not necessarily rely on Nas6. To examine the properties of cognate Rpt3 tail-α2 and Rpt6 tail-α3 interactions in the proteasome under ATP and ADP, we used a previously established, structure-guided disulfide (S-S) crosslinking approach, in which one cysteine is engineered on a specific Rpt C-terminus and the other cysteine is on its cognate α subunit^29^ (Fig. 6a). First, we examined Rpt3 tail-α2 interaction using a set of yeast strains, *rpt3-K428C α2-A79C-HA*_*6*_ and *rpt3-K428C α2-A79C-HA*_*6*_ *nas6*Δ. We isolated endogenous proteasomes from these cells through a 6xHA tag on the α2 subunit and then induced Rpt3-α2 crosslinking by adding the chemical crosslinker, BMOE under ATP or ADP condition (Fig. 6a). In the presence of ATP, Rpt3-α2 crosslinking occurred robustly (Fig. 6a) as evident from a shift in the molecular weight of Rpt3 (50 kDa) to Rpt3-α2 (80 kDa)^29^. Contrarily, Rpt3-α2 crosslinking dramatically diminished under ADP by almost 90% as compared to ATP condition (Fig. 6a,c,d). Nas6 did not noticeably influence Rpt3-α2 crosslinking since Nas6 does not act on fully-formed proteasomes. These results indicate that Rpt3 tail-α2 interaction exhibits strong ATP-dependence in the proteasome.

Next, we examined Rpt6 tail-α3 interaction in the proteasomes isolated from *rpt6-K405C α3-T81C-HA*_*6*_ and *rpt6-K405C α3-T81C-HA*_*6*_ *nas6*Δ cells via α3-HA_6_. Although Rpt6-α3 crosslinking was readily detectable in the presence of ATP (Fig. 6b, left), it was not as robust as Rpt3-α2 crosslinking (Fig. 6c, ATP). In fact, Rpt6-α3 crosslinking was estimated to be approximately 40% less than Rpt3-α2 crosslinking (Fig. 6c, ATP), indicating that Rpt6 tail-α3 interaction in the proteasome holoenzyme is comparatively weaker than Rpt3 tail-α2 interaction. These data are consistent with high-resolution structures of the proteasome holoenzymes, in which the Rpt3 tail but not Rpt6 tail is clearly visualized within its cognate α pockets^4^,^5^. Nas6 did not influence Rpt6-α3 crosslinking. Intriguingly, Rpt6-α3 crosslinking under ADP was readily discernible (Fig. 6b, right), exhibiting 30–40% percent decrease as compared to ATP condition (Fig. 6c,d), indicating that the Rpt6 tail is less nucleotide-dependent than the Rpt3 tail in their CP binding. Together, our data indicate that the Rpt6 tail-α3 interaction in the proteasome holoenzyme occurs with weaker affinity than the Rpt3 tail-α2 interaction under physiological ATP concentration, and is not as nucleotide-dependent as Rpt3 tail-α2 interaction.

Based on our results (Fig. 6a–d) and a joint function of Rpt6 and Rpt3 tails (Figs 1, 2, 3), we predict that decreased Rpt6-α3 interaction in the proteasome may provide relatively weak but consistent affinity to promote Rpt3-α2 interaction. If our prediction is correct, perturbation of the Rpt6 tail as in *rpt6*-Δ*1* cells is expected to negatively affect Rpt3 tail-α2 interaction in the proteasome holoenzyme. Indeed, Rpt3-α2 interaction decreased in *rpt6*-Δ*1* cells as compared to wild-type (Fig. 6e, arrow heads). These data support that Rpt6 tail-α3 binding is important for Rpt3 tail-α2 binding, although the former is comparatively weaker than the latter in the mature proteasome (Fig. 6c, ATP).

**Figure 7:**
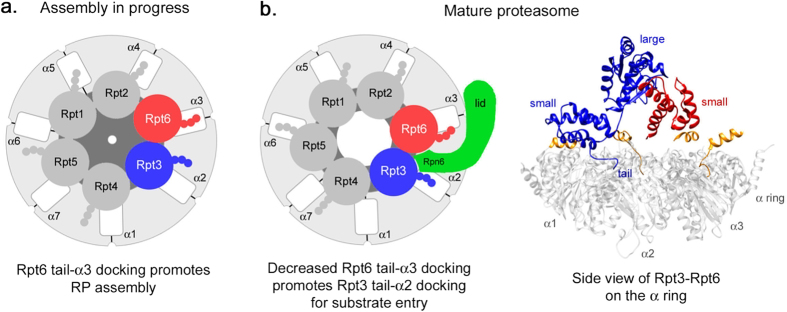
Model of the functional switch of the Rpt6 tail from anchoring for RP assembly into promoting activation of the mature proteasome. (**a**) Cartoon depicting Rpt tail-α pocket interaction during Rpt ring assembly. The Rpt6 tail serves as an anchor by interacting with its cognate α3 pocket to promote Rpt ring assembly^13^,^17^. Rpt6 and Rpt3 subunits are in red and blue, respectively. All other Rpt subunits are in medium gray. Rpt tails are shown as three small spheres. The α subunits are in light gray with white intersubunit pockets. The CP gate is depicted in the center of the α ring and cannot maintain an open configuration (dark gray). (**b**) *Left*, in the mature proteasome, the Rpt6 tail shifts away from the α3 pocket, resulting in a decreased affinity for α3 (Fig. 6). Decreased Rpt6-α3 interaction promotes Rpt3-α2 interaction (Fig. 6) by positioning the Rpt3 tail towards its cognate α2 pocket, thereby facilitating CP gate opening (Fig. 2, see Discussion). Positioning of the Rpt3-Rpt6 dimer at the Rpt ring-CP α ring interface can be stabilized via lid subunit Rpn6 (green), which has been proposed to serve as a molecular clamp and a crucial regulator of proteasome assembly *in vivo*^38^,^45^. All three HbYX-type Rpt proteins (Rpt2, Rpt3 and Rpt5) are in favorable position to interact with their cognate α pockets^4^,^5^. *Right*, UCSF Chimera software^46^ was used to visualize the interaction between Rpt3 and Rpt6 while the Rpt3 tail is docked into its cognate α2 pocket^4^,^5^ within the proteasome (PDB 4CR2^47^). The small AAA domain of Rpt6 (red, small) interacts with the large AAA domain of Rpt3 (large, blue) to form an intersubunit module^37^, which may also contribute to the functional switch of the Rpt6 tail by staying rigid during ATP hydrolysis cycles in the proteasome holoenzyme (see Discussion). For the other Rpt proteins, only their C-terminal tail segments are shown (orange). For clarity, the Rpn6 clamp (**b**), left) and other RP subunits are omitted. The α ring is in light gray. Amino acid residue numbers are: Rpt3 small domain, 343–428; Rpt3 large domain, 177–339; Rpt6 small domain, 320–396.

In summary, our data suggest that the function of the Rpt6 tail switches from single-handedly serving as an anchor during RP assembly^13^,^17^, into jointly acting with the Rpt3 tail to promote gate opening for substrate entry in the proteasome holoenzyme (Fig. 7).

## Discussion

It is well agreed that Rpt C-terminal tails insert into the CP α pockets as a central mechanism to mediate Rpt ring-CP α ring interaction for proteasome assembly and activation^10^,^11^,^13^,^14^,^16^,^19^,^20^,^25^,^29^. In the assembly model, Rpt6 tail docking into α3 pocket has been suggested to position Rpt3-Rpt6 dimer on the CP first, and to incorporate Rpt2-Rpt1 and Rpt5-Rpt4 to form base-CP complex, which then joins with the lid to complete the proteasome holoenzyme^13^,^17^,^18^,^22^,^23^. In the present study, our data suggest that upon proteasome assembly, the Rpt6 tail binds to α3 pocket with decreased affinity, whereas Rpt3 tail binds to α2 with increased affinity (Fig. 6). The anchoring role of the Rpt6 tail on the CP during assembly^13^,^17^ may be relieved as the Rpt3 tail docks into the CP, providing mechanistic insights into apparently transient CP-Rpt6 tail interaction in the proteasome^4^,^5^. A similar mechanism involving ‘affinity switch’ has been reported during CP assembly for its dimeric chaperones Pba1-Pba2 whose C-terminal tails individually insert into α5 and α6 pocket and block RP binding to the CP^33^,^34^. Strong binding affinity of Pba1-Pba2 to the immature CP significantly weakens in the mature CP, thereby allowing the mature CP to associate with the RP.

The affinity switch of the Rpt6 tail to the CP is likely to be driven by the changes in distance between them as the nascent proteasome matures. Because of the 6:7 symmetry mismatch, the orientation between the hexameric Rpt ring and the heptameric CP α ring is tilted and misaligned^29^,^35^,^36^. During assembly, the Rpt6 tail is docked into its cognate α3 pocket^17^ (Fig. 7a). In the mature proteasome, conversely, the Rpt6 tail is positioned away from α3^4^,^5^,^37^. Increased distance between the Rpt6 tail and α3 pocket would result in a decreased affinity of Rpt6 tail-α3 interaction in the proteasome holoenzyme (Figs 6 and 7b). The new position of Rpt6 in the assembled proteasome can be stabilized via its interaction with a lid subunit Rpn6, which contacts the Rpt6-Rpt3 dimer and extends further to bind the CP α ring^5^,^38^. Importantly, our data suggest that a weak Rpt6 tail-CP interaction persists and promotes Rpt3 tail-CP interaction (Figs 6 and 7b). Such a linked change can also be understood from the interaction within the Rpt6-Rpt3 dimer in the Rpt ring. The small AAA domain of Rpt6 and the large AAA domain of Rpt3 form an intersubunit module that remains rigid during ATP hydrolysis cycle in the proteasome holoenzyme^37^. Upon completion of assembly, hydrolysis of an ATP molecule in the Rpt ring is predicted to shift the Rpt6 tail away from α3 pocket but the Rpt3 tail towards α2 pocket, decreasing Rpt6 tail-α3 interaction while enhancing Rpt3 tail-α2 interaction (Figs 6 and 7b). In support of this model, the Rpt6 tail is longer than the Rpt3 tail by 2 amino acids based on the structure-based sequence alignment of Rpt proteins^39^. This would allow the Rpt6 tail to be away from α3 but to weakly interact with it, so as to facilitate the Rpt3 tail to position closely to its cognate α2 pocket. Consistently, substitution of the last residue in Rpt6 does not result in strong phenotypes as the deletion in *rpt6*-Δ*1* cells^17^. Also, lengthening the Rpt6 tail by inserting a single amino acid at the −4 position from the C-terminus negatively affects proteasome assembly and function^13^.

Our data suggest that decreased Rpt6 tail-α3 interaction in the proteasome may support its new role in promoting substrate entry at the RP-CP interface via facilitating Rpt3 tail-α2 interaction. Specificity of Rpt tail-α pocket interaction is critical for maintaining an open configuration of the gate, particularly for Rpt3 whose tail peptide itself prefers non-cognate pockets^11^,^15^,^17^, thereby failing to open the CP gate on its own^11^,^15^. The Rpt3 tail may remain properly bound to its cognate α2 pocket partly via constraints of Rpt6 tail-α3 binding that occurs with low affinity in the mature proteasome (Figs 2 and 6). The Rpt ring-α ring interface forms a narrow axial channel for substrate translocation^6^,^37^. Importantly, Rpt6 and Rpt3 are at a unique location at the Rpt ring-CP α ring interface, where all three subassemblies of the proteasome converge: lid, base, and CP^5^,^38^. At this location, the RP shifts and slides relative to the CP as substrates translocate into the CP in an ATP-dependent manner^6^,^37^. These conformational changes at the RP-CP interface may be facilitated via decreased Rpt6 tail-α3 interaction that is also less ATP-dependent than Rpt3 tail-α2 interaction (Fig. 6). Our data agree with previous studies, which suggest that Rpt6 may have different nucleotide binding properties than other Rpt subunits^20^,^37^.

Together with altered properties of Rpt6 tail-α3 interaction, we identified a novel functional relationship between the Rpt6 tail and Nas6 (Figs 3 and 4). Nas6 binding to Rpt3 provides extensive steric clash against the CP, blocking Rpt3 tail binding to CP^17^,^26^. At the onset of assembly when only the Rpt6 tail anchors onto the CP, Nas6 can remain bound to Rpt3 and prevent premature Rpt3 tail-CP binding. As the role of the Rpt6 tail switches to promote Rpt3 tail-CP binding in the nascent proteasome, Nas6 would further clash against the CP and be expelled (Figs 3 and 5g). The role of Rpt6’s cognate chaperone, Rpn14 remains to be understood. Intriguingly, Rpt6 exhibits conformational dynamics in the C-terminal domain as a potential regulatory mechanism for Rpn14 binding and proteasome assembly^40^. Also, a recently identified chaperone, Adc17 binds to Rpt6 in the N-terminal domain to promote Rpt6-Rpt3 dimerization^41^. While the relationship amongst these events is unknown, the critical role of Rpt6 is further supported by the complex regulation that involves at least three different chaperones, Nas6, Rpn14 and Adc17.

## Methods

### Yeast Strains

All yeast-related procedures were performed as described before^42^. A complete list of yeast strains used in this study is provided in the Table 1.

To construct yeast strains for crosslinking experiments (Fig. 6, Table 1), we isolated genomic DNA from the original strains^29^ to PCR-amplify the entire open reading frame of *α2* or *α3* subunit containing cysteine substitutions, and joined this DNA fragment with a klTRP marker module (pYM3) using a PCR-based strategy^43^ and transformed it into DF5 diploid. Transformants were then sequence-verified, dissected to obtain haploids, and were crossed to *nas6*Δ strain. When the selection markers overlap, all four progenies from tetrad analysis were verified by PCR using the primers specific for a given chromosomal locus to match the marker with the specific gene substitution.

### Antibodies

Antibodies to CP (MCP231, BML-PW8195), Ubiquitin (BML-PW0930), Rpt5 (BML-PW8245), and Rpt3 (BML-PW8250) were purchased from Enzo Life Sciences. Antibodies to Rpt1 and Rpt6 (gifts from Carl Mann), and Rpn8, Rpn12, Rpn14, and Nas6 (gifts from D. Finley) were used. Pgk1 (Invitrogen) was used as a loading control. HRP-conjugated anti-HA antibody (clone 3F10, Roche Life Science) was used to detect HA-tagged α2 and α3.

### Phenotypic Assays

Overnight yeast cultures were diluted into fresh YPD to O.D._600 _= 0.2 and grown until O.D._600 _= 0.8–1.2. Cells were diluted in sterile water to O.D._600 _= 0.4 to prepare a series of 4-fold dilutions in 96-well plates. Cells were spotted onto fresh plates prepared 1 day before the experiments and grown for 2–3 days at indicated temperatures.

### Preparation of Cell Lysates

Yeast cells for biochemical experiments were grown to O.D._600 _= 3–5 and harvested by centrifugation at 3000 × g, washed with cold water, frozen and ground in the presence of liquid nitrogen as described previously^13^,^17^. Cryo-lysates were hydrated with buffer, referred to as the proteasome buffer henceforth (50 mM Tris-HCl, pH 7.5, 5 mM MgCl_2_, 1 mM EDTA, 10% glycerol, and protease inhibitors) that was supplemented with either 2 mM ATP and an ATP regeneration system or 2 mM ADP unless otherwise indicated. Hydrated cryo-lysates were centrifuged at 15,000 rpm for 30 min at 4 °C to obtain whole cell lysates.

### Native PAGE, SDS-PAGE and Immunoblotting

Native PAGE was performed as described before^30^. For in-gel peptidase assay, the native gels were incubated in LLVY-AMC (Suc-Leu-Leu-Val-Tyr-AMC, Bachem) in a buffer (50 mM Tris-HCl, pH 7.5, 5 mM MgCl_2_, 1 mM ATP, 10 μM LLVY-AMC) for 15 min at 30 °C. When indicated, native gels were further incubated with 0.02% SDS for 15 min to induce complete opening of the CP gate^8^. The native gels were imaged using a GENEFLASH (Syngene) system. For quantification of proteasome activities, the colors on native gels were inverted, so that the signals of proteasome activities (RP_2_-CP and RP_1_-CP) are in black and the gel background is in white. For immunoblotting, native gels were transferred to PVDF as described previously^13^,^14^.

For consecutive native PAGE and SDS-PAGE assay, whole cell lysates were first resolved by 3.5% native PAGE, and subjected to an in-gel peptidase assay using LLVY-AMC. The proteasomes were then visualized under UV light, and excised horizontally as a strip. Native gel strips were soaked in 1× SDS Laemmli sample buffer with 2.5% β-ME for 15 min at room temperature and subjected to 12% SDS-PAGE as described previously^14^,^18^.

### Affinity-purification of Proteasome, RP, and CP

Yeast cultures were grown to O.D._600 _= 3–5. Cells were harvested by centrifugation, frozen and ground in the presence of liquid nitrogen. The proteasome, RP and CP were affinity-purified via Protein A tag that is appended to either a lid subunit Rpn11 or a CP subunit Pre1 using IgG resin (MP Biomedicals) following the procedures described previously^44^ except one modification that all buffers contained 10% glycerol throughout the purification. Proteasomal complexes were eluted from the IgG resin using TEV protease (ProTEV, Promega). To obtain IgG resin-bound RP, TEV protease cleavage was omitted.

### Recombinant Chaperone Purification

Nas6 (pJR40) and Rpn14 (pJR56) were expressed and purified from *E. coli* as described previously^26^.

### RP-CP Association Assay

RP was affinity-purified from *nas6*Δ strain (JF6A) and immobilized onto the IgG resin. Resin-bound RP was incubated with affinity-purified CP (2 μg) with or without recombinant Nas6 (0.5 μg) for 30 min at 30 °C in 40 μl proteasome buffer, in the presence of ATP or ADP (2 mM). Resin-bound material was washed twice with 400 μl proteasome buffer containing 150 mM NaCl, and then eluted with TEV protease in 40 μl proteasome buffer. ATP or ADP was included throughout the entire procedures. The eluates were subjected to 12% SDS-PAGE and immunoblotting.

### *In  vitro*  Chaperone  Assay  with  Purified  Proteasome

Proteasomes  were  isolated  via Rpn11-TeV-ProA^44^ (Table 1). Purified proteasomes (37.5 nM) were incubated with recombinant chaperones in 1, 5, and 25-fold molar excess of the proteasome in 16 μl proteasome buffer containing appropriate nucleotides for 30 min at 30 °C. The reactions were subjected to 3.5% native PAGE and immunoblotting.

### Crosslinking

All strains used for crosslinking experiments carry a 6xHA tag that is appended to the C-terminus of the indicated α subunit in their chromosomal loci (Table 1). The crosslinking procedure was essentially performed as described previously^29^. Cryo-lysates were hydrated in the proteasome buffer supplemented with either ATP (1 mM) or ADP (2 mM) as described earlier. Cell lysates were then equalized to 600 μl (approximately 2.5 mg of total cell protein) in the proteasome buffer and incubated with 10 μl of HA-agarose beads (Anti-HA Affinity Matrix, Roche Life Science) on the rotator at 4 °C for 1.5 hr. HA-resin-bound proteasomes were then collected by centrifugation at 15,000 rpm for 30 s at 4 °C, and were washed twice with 400 μl of wash buffer (50 mM Tris-HCl, pH 7.5, 5 mM MgCl_2_, 1 mM EDTA, 10% glycerol, 150 mM NaCl) supplemented with 1 mM ATP or 0.5 mM ADP at 4 °C. To carry out the crosslinking reaction, HA-resin-bound proteasomes were resuspended in 80 μl proteasome buffer containing ATP (1 mM) or ADP (2 mM), and divided equally into two tubes. Under each nucleotide condition, we used one tube for crosslinking reaction by adding 0.1 mM of a chemical crosslinker, BMOE (Bis-Maleimidoethane, Thermo Scientific), and the other tube as a control by adding its solvent, DMF (Sigma-Aldrich). These samples were incubated for 1 hr on the rotator at 4 °C. Crosslinking reactions were quenched by boiling the samples at 95 °C in 55 μl Laemmli 1× sample buffer with 2.5% β-ME, and subjected to 12% SDS-PAGE and immunoblotting.

All biochemical and genetic experiments were conducted at least twice.

## Additional Information

**How to cite this article**: Sokolova, V. *et al*. Proteasome Activation is Mediated via a Functional Switch of the Rpt6 C-terminal Tail Following Chaperone-dependent Assembly. *Sci. Rep*. **5**, 14909; doi: 10.1038/srep14909 (2015).

## Supplementary Material

Supplementary Information

## Figures and Tables

**Table 1:** Strains used in this study.

Strain	Genotype*	Figure	Reference
SUB62	*MAT**a** lys2-801, leu2*–*3, 2*–*112, ura3*–*52, his3*–Δ*200, trp1-1*	1a,b, 2a,c, 3b–d, 4a,b, 5a–c	ref. 48
SP1473A	*MATα rpt3::rpt3*-Δ*1(kanMX6)*	1a,b, 2a,c, 3b–d, 4a,b, 5a–c	
SP1729A	*MAT**a** rpt6::rpt6*-Δ*1(kanMX6)*	1a,b, 2a,c, 3b,d, 4a,b, 5a–c	
SP1785A	*MAT**a** rpt3::rpt3*-Δ*1(kanMX6) rpt6::rpt6*-Δ*1(kanMX6)*	1a,b, 2a,c, 3b,d, 4a,b, 5a–c	
SP1473A	*MAT**a** rpt1::rpt1*-Δ*1(kanMX6)*	1a,b, 2a,c	
SP1475	*MAT**a** rpt2::rpt2*-Δ*1(kanMX6)*	1a,b, 2a,c	
SP1200	*MAT**a** rpt1::rpt1*-Δ*1(kanMX6) rpt2::rpt2*-Δ*1(kanMX6)*	1a,b, 2a,c	
SP1724	*MAT**a** rpt4::rpt4*-Δ*1(kanMX6)*	1a,b, 2a,c, 3c,d	
SP1727A	*MATα rpt5::rpt5*-Δ*1(kanMX6)*	1a,b, 2a,c	
SP1784A	*MAT**a** rpt4::rpt4*-Δ*1(kanMX6) rpt5::rpt5*-Δ*1(kanMX6)*	1a,b, 2a,c	
SP1849B	*MAT**a** rpt3::rpt3*-Δ*1(kanMX6) rpt4::rpt4*-Δ*1(kanMX6)*	3c,d	
SP404	*MATα pre1::PRE1-TEV-ProA(HIS3)*	3a, 5g	ref. 44
SP2230A	*MATα rpt3::rpt3*-Δ*1(kanMX6) pre1::PRE1-TEV-ProA(HIS3)*	3a	
SP1790A	*MATα rpt6::rpt6*-Δ*1(kanMX6) pre1::PRE1-TEV-ProA(HIS3)*	3a	
SP2231A	*MATα rpt3::rpt3*-Δ*1(kanMX6) rpt6::rpt6*-Δ*1(kanMX6) pre1::PRE1-TEV-ProA(HIS3)*	3a	
SP1694A	*MATα nas6::TRP1*	4a,b, 5a–c	
SP1787A	*MATα rpt3::rpt3*-Δ*1(kanMX6) nas6::TRP1*	4a,b, 5a–c	
SP2103	*MAT**a** rpt6::rpt6*-Δ*1(kanMX6) nas6::TRP1*	4a,b, 5a–c	
SP2099A	*MAT**a** rpt3::rpt3*-Δ*1(kanMX6) rpt6::rpt6*-Δ*1(kanMX6) nas6::TRP1*	4a,b, 5a–c	
SP661-1	*MATα rpn11::RPN11-TEV-ProA (HIS3)*	5f	ref. 44
JF6A	*MAT**a** rpn11::RPN11-TEV-ProA (HIS3) nas6::TRP1*	5g	
SP2001B^a^	*MATα rpt3::rpt3-K428C (kanMX6) α2::α2-G79C-HA*_*6*_*(TRP1)*	6a,e	ref. 29
SP2000A^a^	*MAT**a** rpt3::rpt3-K428C (kanMX6) nas6::TRP1 α2::α2-G79C-HA*_*6*_*(TRP1)*	6a	ref. 29
SP1863A^a^	*MATα rpt6::rpt6-K405C (kanMX6) α3::α3-T81C-HA*_*6*_*(TRP1)*	6b	ref. 29
SP1864A^a^	*MAT**a** rpt6::rpt6-K405C (kanMX6) nas6::TRP1 α3::α3-T81C-HA*_*6*_*(TRP1)*	6b	ref. 29
SP2353A^a^	*MATα rpt3::rpt3-K428C (kanMX6) α2::α2-G79C-HA*_*6*_*(TRP1) rpt6::rpt6*-Δ*1(kanMX6)*	6e	ref. 29

SP661-1 and SP404 are MATα strains that derived from sDL135 and sDL133^44^, respectively. Strains were constructed in this study unless otherwise specified.

^*^All strains are congenic to SUB62 background.

^a^These strains derived from ref. 29 (see Methods).
