# Supplementary material for: Proteasome Activation is Mediated via a Functional Switch of the Rpt6 C-terminal Tail Following Chaperone-dependent Assembly
Source: Sci Rep. 2015 Oct 9;5:14909. doi: 10.1038/srep14909 (PMC4598862; doi:10.1038/srep14909)
Supplement: Supplementary Information [file srep14909-s1.pdf]

SUPPLEMENTARY INFORMATION

Proteasome Activation is Mediated via a Functional Switch of the Rpt6 C-terminal Tail Following Chaperone-dependent Assembly

Vladyslava Sokolova[#], Frances Li[#], George Polovin and Soyeon Park*

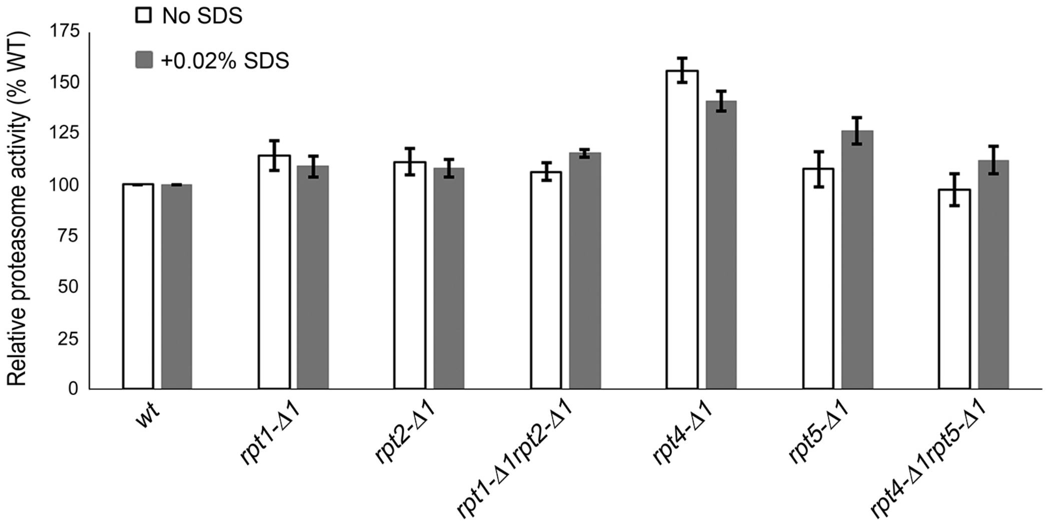

Supplementary Figure 1. Quantification of proteasome activities from Rpt tail mutants. Relative proteasome activities (RP_2-CP and RP_1-CP) from independent experiments (n=6) as in Fig. 2a are shown in mean ± SEM (standard error of the mean). Values of proteasome activities of the indicated cells were quantified using ImageJ software, and divided by the values of wild-type to obtain the relative proteasome activities on the Y axis. Calculations were performed individually for samples without SDS as in Fig. 2a (top panel), and with 0.02% SDS as in Fig. 2a (bottom panel) for each experiment (n=6). Note that the *rpt4-Δ1* proteasomes appear to exhibit increased activities, indicating that CP-Rpt4 tail interaction might prevent aberrant enhancement of gate opening in the proteasome.

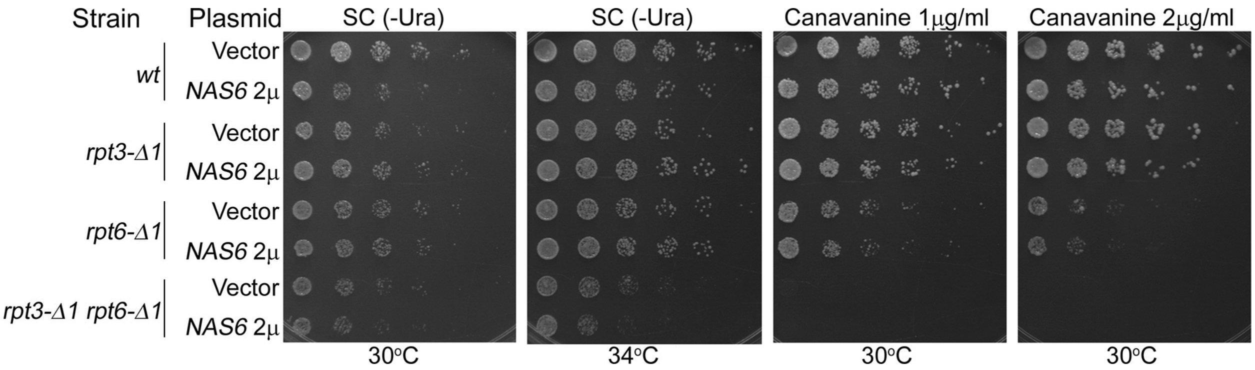

Supplementary Figure 2. Nas6 overexpression does not rescue growth defects of *rpt3-Δ1, rpt6-Δ1* or *rpt3-Δ1rpt6-Δ1* cells. Nas6 was overexpressed from a high-copy 2μ plasmid carrying uracil as a selection marker. Either empty vector or Nas6 plasmid was transformed into the indicated yeast strains. Four-fold serial dilutions of the indicated cells were spotted onto synthetic medium lacking uracil (SC-Ura). Canavanine (an arginine analog) was supplemented to synthetic medium lacking both uracil and arginine. Cells were grown for 2-3 days at indicated temperatures.

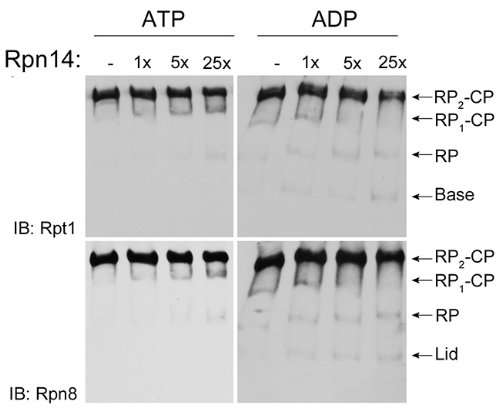

Supplementary Figure 3. Rpn14 does not promote RP dissociation from the proteasome in the presence of ADP. Immunoblotting of native gels showing the effect of Rpn14 on the RP-CP interaction in the proteasome. Affinity-purified proteasomes (0.6 pmol) were mixed with 1, 5, and 25 fold molar excess of recombinant Rpn14 for 30 min at 30°C in the presence of ATP (2 mM) and an ATP regeneration system or ADP (2 mM). The samples were then resolved by 3.5% native gel and immunoblotted for Rpt1 (base subunit) and Rpn8 (lid subunit).
